# Primary effusion lymphoma in an elderly patient effectively treated by lenalidomide: case report and review of literature

**DOI:** 10.1038/bcj.2014.6

**Published:** 2014-03-07

**Authors:** A Antar, H El Hajj, M Jabbour, I Khalifeh, F EL-Merhi, R Mahfouz, A Bazarbachi

**Affiliations:** 1 Division of Hematology and Oncology, Department of Internal Medicine, American University of Beirut Medical Center, Beirut, Lebanon; 2 Department of Pathology and Laboratory Medicine, American University of Beirut Medical Center, Beirut, Lebanon; 3 Department of Radiology, American University of Beirut Medical Center, Beirut, Lebanon

## Abstract

Primary effusion lymphoma (PEL) is a rare aggressive subset of non-Hodgkin B-cell lymphoma. It is caused by Kaposi sarcoma-associated herpesvirus/human herpesvirus type 8 (KSHV/HHV8). It occurs mainly, but not exclusively, in HIV-positive patients. PEL predominantly develops in serous cavities and occasionally in extracavitary regions. PEL carries a very poor prognosis with a median survival time of <6 months. Indeed, currently used treatment modalities such as CHOP chemotherapy are far from achieving complete and sustainable remission. Therefore, there is no clear standard of care established in the treatment of PEL patients, stressing the need for novel-targeted approaches. Here, we have attempted a comprehensive assessment of the treatment of PEL, discussed avant-garde therapies and updated the state of preclinical research with promising clinical applications in the field. These include inhibitors of viral replication, modulators of cell signaling and inflammation, nuclear factor kappa B (NF-κB) and histone deacetylase inhibitors, and recently the combination of arsenic trioxide and interferon-alpha. Some of these targeted therapies have not yet reached clinical studies, although others were used in a few individual case reports with low numbers of patients. We also describe the first case of a 77-year-old, HIV-negative, HHV8-positive patient diagnosed with PEL limited to the pleural and peritoneal cavities. He received lenalidomide 25 mg/day for 21 days every 28 days. Treatment was well tolerated with no side effects. He rapidly improved after 1 month of treatment and progressively achieved complete remission persistent after 18 months of therapy. We believe that this review will bridge an important gap between classical chemotherapy and modern approaches of targeted therapy. Finally, our findings warrant further evaluation of lenalidomide in future prospective clinical studies.

## Introduction

Primary effusion lymphoma (PEL) is a rare aggressive non-Hodgkin B-cell lymphoma that was first described in 1989 as body cavity-based lymphomas.^1, 2^ It is caused by Kaposi sarcoma-associated herpesvirus/human herpesvirus type 8 (KSHV/HHV8)^3, 4, 5^ and accounts for ∼3% of AIDS-related lymphomas.^6^ It occurs mainly, but not exclusively, in HIV-positive patients, and less frequently in other groups such as elderly patients or organ transplantation recipients.^7, 8, 9^ The majority of PELs occur exclusively as lymphomatous effusions in serous cavities such as pleural, pericardial or abdominal cavities.^10^

PEL is generally resistant to chemotherapy and carries a dismal prognosis with a median survival of around 6 months.^11, 12, 13, 14, 15, 16^ Given its rarity, there are only very few observational series of patients with PEL, and very few prospective trials testing chemotherapy, antiviral therapy or targeted therapy in that setting. Therefore, there is no clear standard of care established in the treatment of PEL patients.

Lenalidomide is an immunomodulatory drug that is commonly used to treat newly diagnosed and relapsed multiple myeloma^17^ as well as a variety of hematological malignancies such as chronic lymphocytic leukemia, mantle cell and diffuse large B-cell lymphomas and myelodysplasias.^18, 19, 20, 21, 22, 23, 24^ Furthermore, lenalidomide was successfully used to treat three patients with advanced refractory Kaposi sarcoma.^25^ It exerts its antitumor action through various mechanisms such as activation of the immune system, inhibition of angiogenesis and direct antineoplastic effects.^26^

## Case presentation

**Figure 1:**
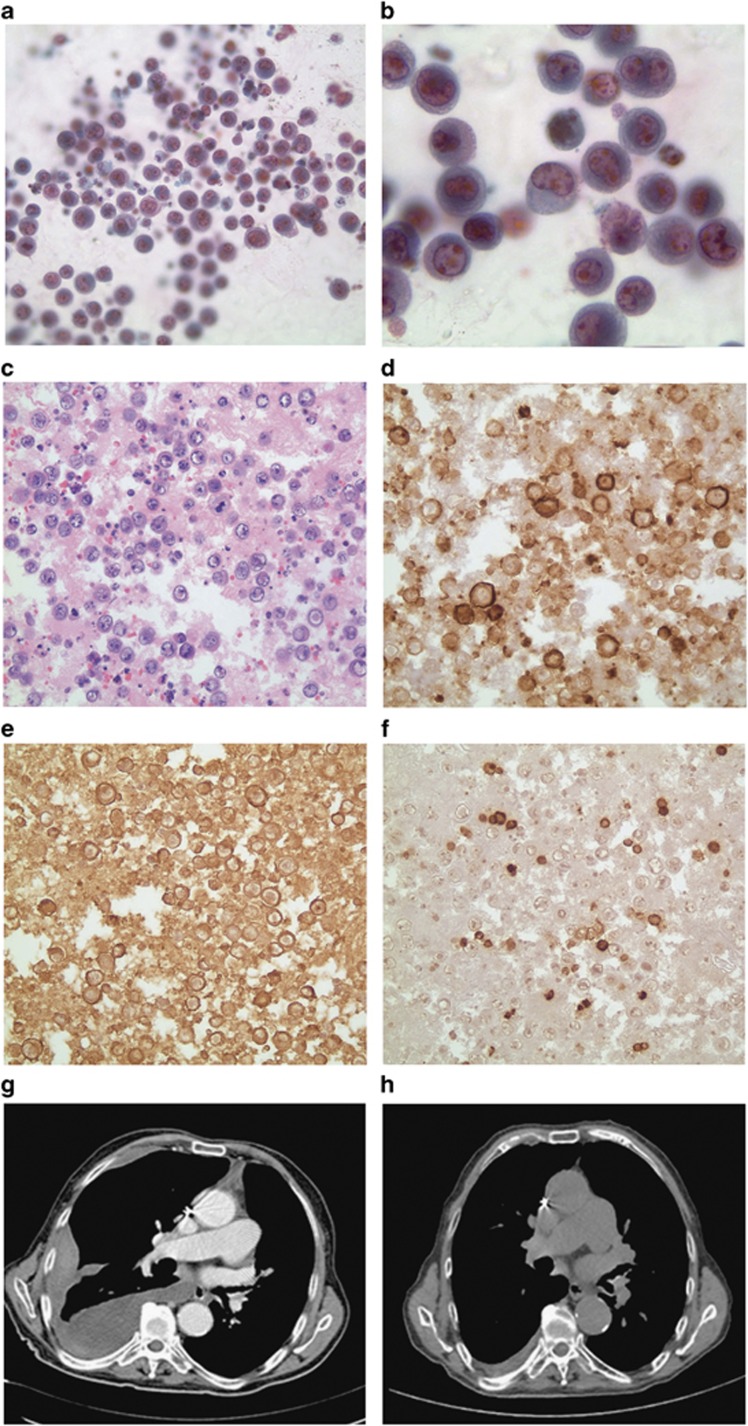
Diagnostic features and follow-up in a PEL patient. (**a**, **b**) Pleural fluid with hypercellular areas composed of discohesive, large, atypical cells characterized by a high nucleocytoplasmic ratio, occasional nuclear bilobation and eccentric nuclei with multiple peripheral nucleoli reminiscent of atypical plasma cells (*magnification:* **a** *× 400 and* **b** *× 1000*). (**c**–**f**) Pleural fluid cell block (H&E stain: **c**; immunohistochemistry **d**–**f**; magnification × 400) showing the discohesive atypical cells (**c**), expressing plasma cell marker CD38 (**d**) and EMA (**e**) but negative for CD45 (**f**). (**g**, **h**) Computed Tomography axial single slice showing pretreatment reaccumulation of the right pleural fluid after successful drainage (**g**) and significant improvement of the right pleural fluid after 8 months of treatment by lenalidomide (**h**).

A 77-year-old man presented with a 2-month history of increasing abdominal girth. The patient had a history of coronary artery disease, congestive heart failure and brain surgery for intracerebral hemorrhage. No history of malignancy, HIV, hepatitis B or hepatitis C infection was noted. Computed tomography (CT) scan of abdomen and pelvis showed a large cystic peritoneal mass sizing up to 30–35 cm in greatest dimension. He underwent a surgical resection of the cystic lesion that turned out to be a chronically inflamed fibromembranous adipose tissue. Two months later, he was admitted because of severe dyspnea. CT scan of the chest and PET-CT scan revealed bilateral pleural effusion, without evidence of abnormal metabolic uptake. Bilateral pig tails were inserted with successful drainage of the effusion. Cytopathology of pleural fluid revealed a clone of malignant lymphoid cells with high N/C ratio, multiple nucleoli and frequent mitosis (Figures 1a and b). By immunohistochemistry, the cells were positive for CD38 and EMA only, and negative for CD45, CD20, CD3, CD43, PAX-5, CD34, CD68, CD117, CD30, TdT, myeloperoxidase, S-100 and CKAE1/3 (Figures 1c–f and not shown). Flow cytometry on the pleural fluid revealed positivity for CD38 and EMA whereas CD138, CD16 and CD56 were negative. Overall, the clinical and pathological features were consistent with PEL. Moreover, a review of pathology of the previous abdominal cyst was consistent with the same diagnosis. Bone marrow biopsy was negative for malignancy. HHV8 serology was positive. HIV serology was negative. Repeated CT scan of the chest after 1 week of pig tail removal showed a significant reaccumulation of the effusion, notably on the right side (Figure 1g).

Because of the patient age and clinical condition, he was started on lenalidomide 25 mg p.o. daily for 21 days every 28 days for each cycle. He had a significant improvement of his dyspnea after the first cycle. Monthly chest X rays and CT scan of chest after eight cycles of treatment showed a major decrease in the right pleural effusion with complete resolution of the left pleural effusion (Figure 1h). Treatment was relatively well tolerated and no side effects were noted. He has been on lenalidomide for 18 months so far, without any symptoms or evidence of disease progression.

## Diagnosis of PEL

The diagnosis of PEL is based on the combination of clinical features, morphology, immunophenotype, virology and molecular findings. The cytological preparations (for example, smears, cytospin and cell block) of the malignant effusion constitute the main material for pathologic examination and diagnosis. The cytologic features are usually large cells with moderate to abundant basophilic cytoplasm, round to irregular nuclei and prominent nucleoli. The cells show a range in appearance from immunoblastic to plasmablastic to anaplastic.^27^ Immunophenotypically, and although of B-cell origin, PEL cells express leukocyte common antigen (CD45) and post-germinal center B cell/plasma cell-associated antigens (CD38, CD138) suggesting a late stage of B-cell differentiation,^28, 29^ but classic B-cell markers (CD19, CD20, CD79a) and T-cell markers (CD3, CD4, CD8) are absent.^29, 30, 31^ In more detail and among 61 cases of PEL reported in the literature, 93% were CD45 positive, 73% were CD30 positive, and finally, T- and B-cell markers were expressed in 4.9 and 1.6%, respectively.^32^ The HHV8 gene expression pattern in PEL cells is mostly latent with expression of LANA-1/2, kaposin and cellular homologs such as v-cyclin, v-FLIP, and to a lesser extent v-IL-6 (ref. 28). PEL cells are commonly co-infected by EBV^10^ that can be specifically detected by *in situ* hybridization for EBV RNA. However, EBV latent membrane protein 1 staining is negative until reactivation.^33, 34^ Molecular analysis usually demonstrates the post-germinal center B-cell origin of PEL by revealing a clonal immunoglobulin gene rearrangements and somatic hypermutation.^35^

## Treatment of PEL

### Chemotherapy

Boulanger *et al.*^36^ described the largest series of PEL patients (*n*=28) with a median follow-up of 3.8 years. This multicenter retrospective clinical series showed a median survival of 6.2 months and a 1-year overall survival rate of 39.3%. No standard treatment is currently available for PEL. The data from six large and most recent PEL published series are summarized in Table 1. The backbone therapy in all these series is CHOP-based chemotherapy (cyclophosphamide, doxorubicin, vincristine, and prednisone). Some patients received more intensive chemotherapy by adding methotrexate to CHOP or by increasing the dose intensity (ACVBP).^12, 13, 37^ Complete remission was achieved in 43% when CHOP-like chemotherapy was used in one study^11^ and 57% and 67%, respectively, when more intensive regimen (CHOP-like+MTX or ACVBP) was used in other studies.^13, 37^ Although the intensive chemotherapy seems to be more effective, most patients cannot tolerate it probably because of the low CD4+ cell count and/or poor performance status in HIV-positive patients and the old age in HIV-negative patients. Moreover, methotrexate tends to accumulate in the effusions, which delayed its clearance and increased toxicity.^38, 39^ Although methotrexate accumulation in serous effusions might be beneficial to patients with PEL, it should be used with caution using intensive hydration, daily monitoring of methotrexate level and leucovorin rescue maintenance until complete methotrexate elimination. Otherwise, methotrexate can be associated with severe systemic toxicity.^40^

### Stem cell transplantation

High-dose chemotherapy and autologous stem cell transplant (ASCT) have been described in two case reports.^41, 42^ The first case was an HIV-negative patient with relapsed PEL who was successfully treated by ASCT,^41^ and the second case was an HIV-positive PEL who had an early progression after ASCT.^42^ On the basis of extrapolation from other studies in HIV-related lymphomas,^43^ ASCT can be considered in patients with relapsed PEL who respond to salvage chemotherapy. However, ASCT as a consolidation therapy in first-line complete remission patients is not supported by any evidence so far. In addition, one case of successful allogeneic stem cell transplantation has been reported in an HIV-positive PEL patient.^44^

### Antiviral therapy

In the pre-HAART era and despite the use of the same chemotherapy regimens as today, the outcome of HIV-positive PEL patients was dismal.^14, 15^ Indeed, the absence of HAART before PEL diagnosis was associated with poor outcome in a multivariate analysis.^36^ In addition, complete remissions have been reported after treatment of PEL patients by HAART alone.^45, 46, 47^ Hence, it is recommended to continue or initiate HAART as a part of supportive therapy in all HIV-positive PEL patients when treatment is started.

Antitumor activity of antiviral therapy against HHV8-associated PEL, whether related or not to HIV, has been reported in two studies that described prolonged complete remission after the intracavitary administration of cidofovir.^48, 49^ Finally, one PEL patient showed a complete response when treated with azidothymidine (AZT) and interferon-alpha (IFN-α).^50^ Cells from this patient as well as PEL cell lines showed that the pro-apoptotic effect of this combination is mediated by a concomitant activation of tumor necrosis factor-related apoptosis-inducing ligand and nuclear factor kappa B (NF-κB) inhibition.^50^ This result was consistent with the prolonged survival of PEL mice treated with AZT and IFN-α.^51^

### Targeted therapy

PEL tumor cells that are latently infected by KSHV/HHV8 display constitutive activity of many signaling pathways for growth and survival, including the NF-κB, JAK/STAT (signal transducer and activator of transcription) and phosphoinositide-3-kinase (PI3K)/AKT pathways.^52, 53, 54^ Several published preclinical studies on targeted therapy are shown in Tables 2 and 3. Since NF-κB activation seems to be a key player in PEL oncogenesis, the antitumor effect of proteasome inhibitors such as bortezomib was investigated in few preclinical trials and very few case reports.^16, 52, 53, 55, 56, 57, 58, 59, 60, 61, 62^ These drugs induce apoptosis of PEL cell lines *in vitro* and preclinical responses *in vivo* in xenograft PEL mouse models. In addition, a combination of proteasome inhibitor and chemotherapy^60^ or histone deacetylase inhibitor^63^ revealed a synergistic preclinical activity. Similarly, promising preclinical results were reported with multiple NF-κB inhibitors such as Berberine, diethyldithiocarbamate, cepharanthine and heat-shock protein 90 (refs. 37,54,55,56). Unfortunately, despite this promising preclinical efficacy of bortezomib, treatment of three chemotherapy refractory PEL patients with this drug alone or in combination with chemotherapy showed no improvement.^64^

Targeting the PI3-kinase pathway^57, 65^ or the JAK/STAT pathway using a JAK2 inhibitor (tyrphostin AG490), or a dominant-negative STAT3-expressing vector, induced apoptosis of PEL cell lines.^66^ However, disappointing results with rapid resistance was reported with rapamycin (Sirolimus, St Louis, MO, USA), an inhibitor of mammalian target of rapamycin (mTOR) signaling.^58, 67^ In addition, the anti-CD30/drug conjugate brentuximab vedotin (Adcetris, Bothell, WA, USA) showed promising preclinical anti-PEL effects.^68^ Finally, in HHV8-positive PEL cell lines, we demonstrated that the combination of arsenic trioxide and IFN inhibits growth and NF-κB activation and induces caspase-dependent apoptosis.^61^ Recently, we showed that in a preclinical NOD/SCID mouse model, this combination downregulates the latent viral transcripts LANA-1, v-FLIP and v-Cyc in PEL cells derived from malignant ascites, decreases the peritoneal volume and synergistically increases survival of PEL mice.^69^

## Conclusion

PEL still carries a dismal prognosis and the currently used treatment modalities are yet far from achieving complete and sustainable remission. On the basis of recent preclinical data and translational studies, several new-targeted therapies are being explored. Treatment with the second generation immunomodulatory drug lenalidomide has never been reported in PEL patients. The dramatic and prolonged efficacy of lenalidomide in the case of an elderly patient with PEL warrants testing this novel agent in prospective studies.

## Figures and Tables

**Table 1:** Clinical series of primary effusion lymphoma patients

	*Boulanger et al.*^36^	*Simonelli et al.*^11^	*Boulanger et al.*^13^	*Boulanger et al.*^12^	*Valencia et al.*^14^	*Nador et al.*^15^
Number of patients	28	11	7	12	7	15
Male	96%	90%	100%	100%	86%	100%
HIV positive	100%	100%	100%	100%	100%	87%
EBV co-infection	72%	n/a	57%	92%	—	93%
History of Kaposi sarcoma	67%	27%	71%	50%	43%	33%
Extra-cavitary disease	43%	45%	71%	42%	0%	13%
HAART	78%	72%	71%	50%	14%	0%
CHOP-like chemotherapy	36%	72%	0%	50%	85%	n/a
CR rate	10%	43%	n/a	17%	n/a	n/a
CHOP-like +methotrexate	35%	0%	100%	17%	0%	n/a
CR rate	70%	n/a	57%	50%	n/a	n/a
ACVBP	8%	0%	0%	8%	0%	n/a
CR rate	50%	n/a	n/a	0%	n/a	n/a
Median overall survival	6.2 months	6 months	9 months	5 months	2 months	4 months

Abbreviations: ACVBP, doxorubicin, cyclophosphamide, vindesine, bleomycin, prednisone; CHOP, cyclophosphamide, doxorubicin, vincristine, and prednisone; CR, complete remission; EBV, Epstein-Barr virus; HAART, highly active antiretroviral therapy; n/a, not applicable.

**Table 2:** Preclinical studies in primary effusion lymphoma targeting the proteasome and/or the NF-κB pathway

*Study*	*Agent*	*Activity*	*Model*	*Key findings*
El Hajj *et al.*^69^	Combination of arsenic trioxide and IFN-α	NF-κB inhibition; antiviral activity	*In vitro:* PEL cell lines; *in vivo*: xenograft mouse model	Dramatic inhibition of proliferation, induction of apoptosis and downregulation of the latent viral transcripts LANA-1, v-FLIP and v-Cyc; decrease in the peritoneal volume and prolonged survival of PEL mice
Bhatt *et al.*^63^	Proteasome/HDAC inhibitor	Proteasome and NF-κB inhibition; histone deacetylase inhibition	*In vivo*: xenograft mouse model	Extensive apoptosis and significant survival advantage in PEL-bearing mice
Goto *et al.*^54^	Berberine (isoquinoline alkaloid)	NF-κB inhibition	*In vitro*: PEL cell lines; *in vivo*: xenograft mouse model	Cell death by blocking the NF-κB pathway in PEL cells; antiretroviral activity
Matsuno *et al.*^37^	Diethyldithio-carbamate (DDTC)	NF-κB inhibition	*In vivo*: xenograft mouse model	Amelioration of PEL symptoms
Higashi *et al.*^56^	Heat-shock protein 90 inhibitors	NF-κB inhibition	*In vitro*: PEL cell lines	Induction of apoptosis in PEL cell lines
Saji *et al.*^62^	MG132, lactacystin, proteasome inh 1	Proteasome and NF-κB inhibition	*In vitro*: PEL cell lines	Inhibition of proliferation and induction of apoptosis in PEL cells
Sarosiek *et al.*^16^	Bortezomib	Proteasome and NF-κB inhibition	*In vivo*: xenograft mouse model	PEL remission and increase of overall survival in PEL-treated mice
Hussain *et al.*^53^	Proteasome inhibitor: MG-132	Proteasome and NF-κB inhibition	*In vitro*: PEL cell lines	Apoptosis of PEL cells *via* downregulation of SKP2 leading to accumulation of p27Kip1
Takahashi *et al.*^55^	Biscoclaurine alkaloid cepharanthine (CEP)	NF-κB inhibition	*In vitro:* PEL cell lines; *In vivo*: xenograft mouse model	No significant systemic toxicity in this model; dose-dependent inhibition of proliferation and apoptosis of PEL cell lines
Abou-Merhi *et al.*^61^	Bortezomib; combination of arsenic and IFN-α	Proteasome and NF-κB inhibition	*In vitro*: PEL cell lines	Dramatic inhibition of cell proliferation and induction of apoptosis
Matta *et al.*^52^	Bortezomib	Proteasome and NF-κB inhibition	*In vitro*: PEL cell lines	More cytotoxicity against PEL cells than against cell lines derived from multiple myeloma
An *et al.*^60^	Bortezomib ±chemotherapy (doxorubicin and taxol)	Proteasome and NF-κB inhibition	*In vitro*: PEL cell lines	TRAIL-induced death, inhibition of cell growth and induction of apoptosis in PEL cells; synergy with chemotherapy

Abbreviations: HDAC, histone deacetylase; IFN-α, interferon-alpha; NF-κB, nuclear factor kappa B; PEL, primary effusion lymphoma; TRAIL, tumor necrosis factor-related apoptosis inducing ligand.

**Table 3:** Preclinical studies in primary effusion lymphoma other than proteasome inhibition

*Study*	*Agent*	*Activity*	*Model*	*Key findings*
*Targeting PI3-kinase pathway*				
Hussain *et al.*^53^	Bay11-7085 ±LY294002	NF-κB±PI3-K inhibition	*In vitro*: PEL cell lines	Synergistic apoptotic responses in PEL cells
Uddin *et al.*^65^	LY294002	PI3-kinase inhibition	*In vitro*: PEL cell lines	Apoptosis in all PEL cell lines studied except BCP1
				
*Targeting mTOR pathway*				
Gasperini *et al.*^67^	Rapamycin (sirolimus)	mTOR inhibition	*In vitro*: PEL cell lines; *in vivo*: xenograft mouse model	No eradication of PEL; after an initial response, development of resistance in PEL cells treated with rapamycin
Sin *et al.*^58^	Rapamycin (sirolimus)	mTOR inhibition	*In vitro*: PEL cell lines; *in vivo*: xenograft mouse model	Inhibition of PEL growth in culture; delay of PEL progression *in vivo*
				
*Targeting STAT3 pathway*				
Aoki *et al.*^66^	Dominant-negative STAT3 vector; tyrphostin AG490	STAT3 inhibition Jak2 inhibition	*In vitro:* PEL cell lines	Induction of apoptosis and decrease of survivin expression in PEL after inhibition of STAT3 signaling
				
*Antiviral±IFN-α*				
Fujimuro *et al.*^70^	Gancyclovir (purine nucleoside analogs)	Antiviral activity	*In vitro*: PEL cell lines	Cytotoxic activity toward KSHV-infected PEL cells treated with gancyclovir but not with acyclovir
Wu *et al.*^51^	AZT and IFN-α	Antiviral activity	*In vivo*: xenograft mouse model	Increased survival of PEL mice
Lee *et al.*^59^	AZT and IFN-α	Antiviral activity	*In vitro*: PEL cell lines	Minimal sensitivity to AZT alone but synergy with IFN-α
				
*Miscellaneous*				
Goto *et al.*^71^	Zoledronic acid	Induction of Vg9Vd2 T cells	*In vitro*: PEL cell lines; *in vivo*: xenograft mouse model	Inhibition of growth of PEL cells and improved survival of PEL mice
Paul *et al.*^72^	Nimesulide	COX-2 inhibitor	*In vitro*: PEL cell lines	Proliferation arrest
Bhatt *et al.*^68^	Brentuximab vedotin	Anti-CD30 monoclonal and drug conjugate	*In vitro*: PEL cell lines; *in vivo*: xenograft mouse model	Proliferation arrest in PEL cell lines; prolonged survival in PEL mice
Lan *et al.*^73^	Gamma-secretase inhibitor (GSI)	Intracellular activated Notch1 (ICN) blockage	*In vivo*: direct xenograft model	Delay of the onset of tumorigenesis of treated PEL mice

Abbreviations: AZT, azidothymidine; HDAC, histone deacetylase; IFN-α, interferon-alpha; KSVH, Kaposi sarcoma-associated herpesvirus; mTOR, mammalian target of rapamycin; NF-κB, nuclear factor kappa B; PEL, primary effusion lymphoma; PI3-K, phosphoinositide-3-kinase; STAT3, signal transducer and activator of transcription 3.
